# Mysterious *Leishmania martiniquensis* parasites and their relatives of the subgenus *Mundinia*: Emerging pathogens reshaping our understanding of leishmaniases

**DOI:** 10.1371/journal.ppat.1014057

**Published:** 2026-03-13

**Authors:** Petr Volf, Jovana Sadlova

**Affiliations:** Department of Parasitology, Faculty of Science, Charles University, Prague, Czechia; Institute of Parasitology, Biology Centre, Czech Academy of Sciences, CZECHIA

Leishmaniases have traditionally been defined by a stable triad: transmission by phlebotomine sand flies, geographically restricted parasite species, and well-established, typically zoonotic reservoirs. This framework has shaped our understanding of *Leishmania* biology, epidemiology, and control for decades. *Leishmania martiniquensis* fundamentally challenges this paradigm. Since its initial recognition in the Caribbean, this species has been detected in regions as distant as Southeast Asia, Central Europe, and the Americas, often in the absence of clearly identifiable reservoirs or confirmed sand fly vectors. Together with other members of the recently established subgenus *Mundinia*, *L. martiniquensis* challenges core assumptions about what defines a *Leishmania* parasite. Its apparent intercontinental distribution strongly suggests anthropogenic spread, plausibly mediated by cosmopolitan rodents such as rats, which are repeatedly found to harbor the parasite.

## *Mundinia*: An ancient lineage rediscovered

**Fig 1:**
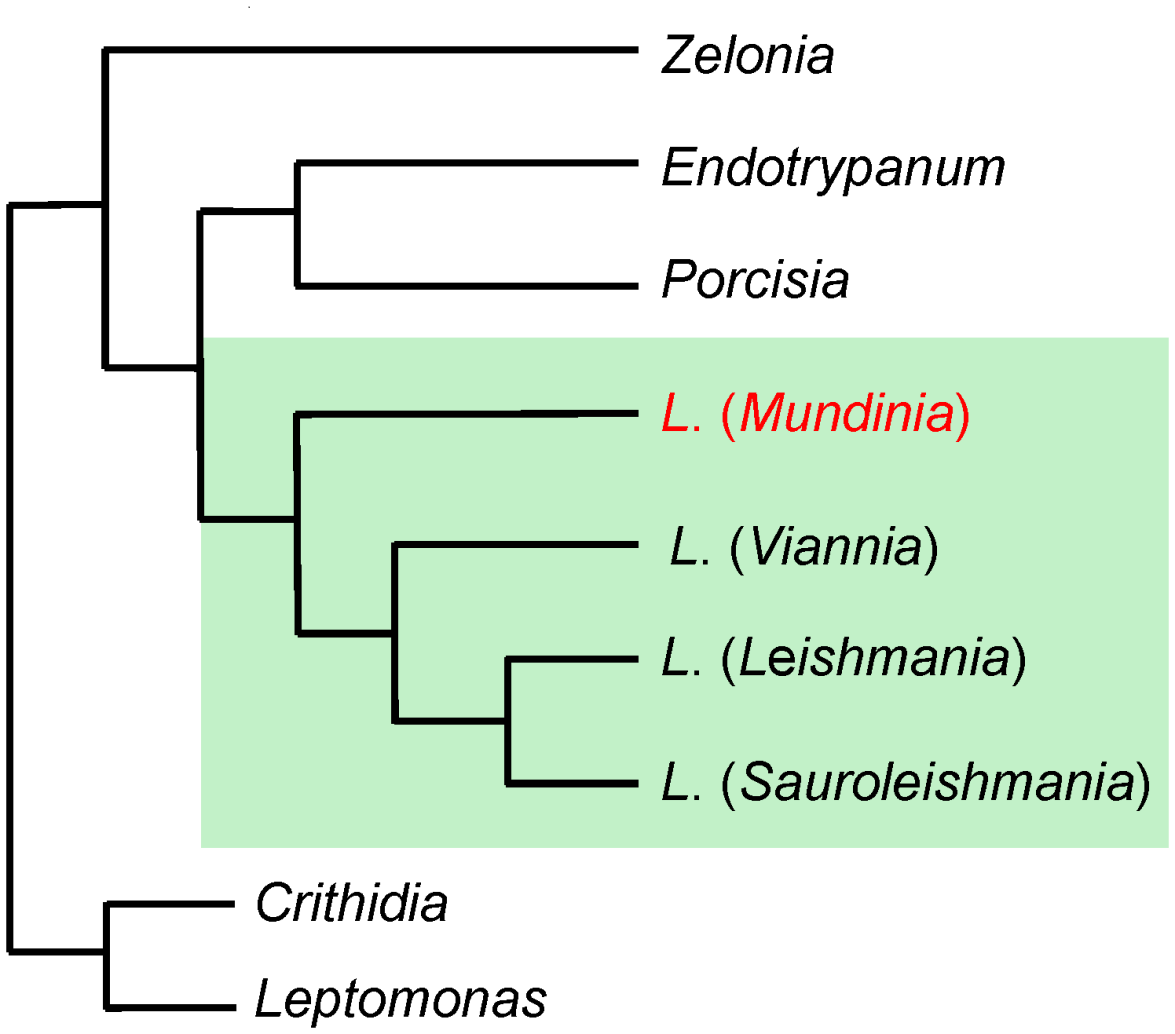
Schematic representation of the phylogenetic position of the subgenus *Mundinia* in relation to other heteroxenous trypanosomatid genera of the subfamily Leishmaniinae, with *Crithidia* and *Leptomonas* as outgroups, according to [1].

The subgenus *Mundinia* was formally established in 2016 to accommodate a group of parasites previously classified within the *Leishmania enriettii* complex [1]. Phylogenomic analyses consistently place *Mundinia* at the base of the *Leishmania* phylogeny, identifying it as the earliest-diverging lineage of the genus (Fig 1) [1–4]. This deep evolutionary position suggests a long history of co-evolution with vertebrate hosts and insect vectors, likely predating the diversification of the better-characterized subgenera *Leishmania* and *Viannia*.

Paradoxically, despite their ancient origin, most *Mundinia* species have come to scientific attention only within the past two decades. Several were discovered retrospectively, hidden among misidentified isolates or archived material [4], while others were recognized through investigations of unexpected clinical or veterinary cases [5–8]. This pattern strongly suggests that *Mundinia* parasites are not newly emerging but rather long-established organisms that have largely escaped detection. Their historical invisibility likely reflects a combination of low pathogenicity, asymptomatic persistence in natural hosts, and transmission cycles that fall outside standard surveillance frameworks designed for classical sand fly–borne leishmaniases.

## A parasite without borders

**Fig 2:**
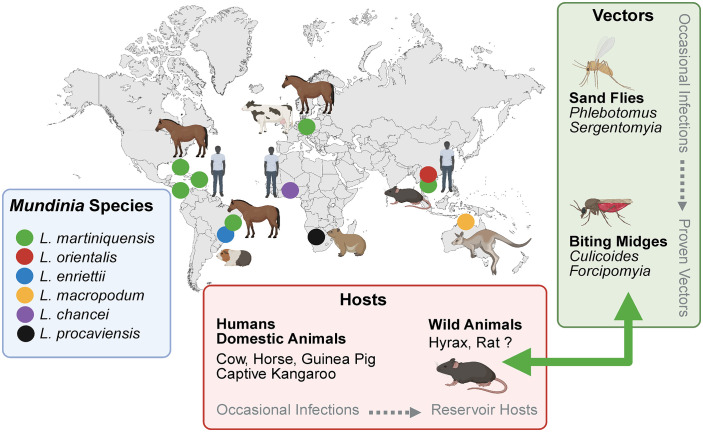
*Leishmania martiniquensis* detected across multiple continents, with rodents as likely anthropogenic dispersal hosts; biting midges as vectors and sand flies occasionally infected. Other *Mundinia* species display strikingly restricted geographic distributions. Created in BioRender. SÁDLOVÁ, J. (2026) https://BioRender.com/er1wbcb.

Most *Mundinia* species display strikingly restricted geographic distributions, typically confined to a single country or region (Fig 2). In this context, *L. martiniquensis* stands out as a conspicuous outlier. Autochthonous infections have been documented across multiple continents, including Southeast Asia, Central Europe, the Americas, and the Caribbean [5–8], affecting both humans and domestic animals. Such intercontinental distribution is difficult to reconcile with the canonical ecology of leishmaniasis, particularly in the absence of a defined sylvatic cycle or a confirmed vector.

Unlike *Leishmania infantum*, whose global spread can be readily explained by long-standing zoonotic reservoirs [9], documented infections with *L. martiniquensis* are sporadic and largely associated with humans or domestic animals, which are unlikely to sustain long-term transmission cycles. However, *L. martiniquensis* has been repeatedly detected in commensal rodents, particularly black rats (*Rattus rattus*) [10,11], implicating these cosmopolitan species as potential agents of anthropogenic dispersal. The repeated identification of autochthonous cases in temperate regions further underscores that *L. martiniquensis* is not merely an imported curiosity, but a parasite capable of establishing local transmission wherever ecological conditions permit.

## The missing reservoirs: Sources, sinks, and silence

A defining feature of classical leishmaniasis is the presence of stable reservoir hosts that sustain transmission independently of human infection. For *Mundinia* parasites, such reservoirs remain largely elusive. Except for *Leishmania procaviensis*, which appears to persist asymptomatically in rock hyraxes [2], convincing evidence for long-term reservoir hosts is lacking across the subgenus. Infections with *L. martiniquensis* in humans and domestic animals are typically sporadic, making it unlikely that these hosts act as epidemiologically relevant sources of infection.

Field studies in endemic areas have detected *Mundinia* DNA in a range of vertebrates, particularly rodents [10,11]. However, parasite detection alone is insufficient to establish reservoir competence. The distinction between “sources” and “sinks” of infection is critical: parasites may transiently infect a host without contributing to onward transmission [9]. Therefore, laboratory experiments on the capacity of rodents to harbor the parasite long-term and their infectiousness to vectors must be experimentally proven. The scarcity of overt disease, combined with low parasite burdens and potential long-term persistence, makes true reservoirs difficult to identify using conventional approaches.

These observations support the hypothesis that *Mundinia* parasites have evolved highly balanced relationships with their natural hosts, resulting in predominantly asymptomatic infections that remain largely invisible to clinical and veterinary surveillance. In such systems, disease may represent occasional spillover rather than the norm, complicating efforts to reconstruct transmission cycles. In the *Leishmania* subgenus, meta-analysis of xenodiagnostic studies and laboratory experiments have revealed a significant role of asymptomatic hosts in the transmission, and experiments on the natural *L. major*—*Meriones shawi*—*Phlebotomus papatasi* model have shown that a surprisingly low infectious dose of 2–10 amastigotes is sufficient to infect the vector [12,13]. Therefore, current knowledge indicates that symptomatic disease and high parasite burdens are not indispensable for transmission. A good parallel is also the subgenus *Sauroleishmania*, in which, as far as is known, infections in reptiles are also largely asymptomatic.

## Rethinking host competence: Insights from experimental models

Experimental infections have provided critical insights also into the biology and host specificity for *Mundinia* parasites. Classical laboratory models widely used for cutaneous and visceral leishmaniasis, including BALB/c mice and guinea pigs, are poorly suited for most *Mundinia* species, typically exhibiting resistance or only transient infections [14–17]. Early failures in these models suggested limited host compatibility, but subsequent studies have revealed a more complex and informative pattern.

Chinese hamsters and steppe lemmings inoculated intradermally with stationary-phase promastigotes support long-term infections with multiple *Mundinia* species, including *L. martiniquensis*. Notably, Chinese hamsters can harbor parasites without overt clinical signs while remaining infectious to insect vectors, closely mirroring the hypothesized natural reservoir state. In contrast, steppe lemmings develop disseminated infections with severe pathology when infected with human-pathogenic species, illustrating a spectrum of disease outcomes driven by host–parasite compatibility [16].

Together, these findings indicate that *Mundinia* parasites are finely adapted to a narrow range of hosts in which they persist silently and efficiently. Disease thus appears to be a consequence of host mismatch rather than a central feature of the parasite’s life history. Rodents emerge as particularly compelling candidates for natural reservoir hosts, highlighting the critical role of experimental studies in defining host competence and reconstructing transmission cycles.

## When sand flies are not involved

Perhaps the most paradigm-challenging aspect of *Mundinia* biology concerns their insect vectors. Transmission of human-pathogenic *Leishmania* has long been regarded as the exclusive domain of phlebotomine sand flies (Diptera: Psychodidae), a criterion so fundamental that it has effectively defined the genus. For *Mundinia* parasites, however, this assumption no longer holds. Field surveys in endemic areas have occasionally detected *Mundinia* DNA in sand flies, including *Phlebotomus stantoni* and several *Sergentomyia* species [18]. But such infections were rare and provided little evidence of functional vector capacity.

In contrast, biting midges (Diptera: Ceratopogonidae) have emerged as unexpected but plausible alternative vectors. In Australia, where investigations first extended beyond sand flies, late-stage infections and metacyclic forms were observed in *Forcipomyia* midges [19]. Subsequent experimental studies demonstrated that multiple *Mundinia* species can complete development in midges, colonize their stomodeal valve, and transmit to vertebrate hosts, whereas parallel infections in sand flies were limited or abortive [15,20–23].

Field data further supports this apparent vector shift. In Southeast Asia, natural infections have been documented in non-engorged midges (*Culicoides mahasarakhamense*, *C. peregrinus*, *C. oxystoma*, among others) collected in proximity to human cases, including settings with co-circulation of *L. orientalis* and other trypanosomatids [18]. Altogether, these findings meet most classical criteria for vector competence and provide robust evidence for non-sand fly transmission within the genus *Leishmania*. Confirmation of biting midges as natural vectors would fundamentally alter our understanding of leishmaniases ecology and the defining biological features of the genus.

## Why *Mundinia* matter

*Mundinia* parasites, and *L. martiniquensis* in particular, occupy an uneasy position at the margins of established paradigms. Their ancient evolutionary origin contrasts sharply with their recent recognition; their apparent global distribution defies expectations in the absence of clearly defined reservoirs; and their ability to develop in insect vectors other than sand flies challenges one of the fundamental features of *Leishmania* biology.

Beyond their immediate relevance to parasitology, *Mundinia* parasites highlight the need for integrated approaches that combine field studies, laboratory experiments, and molecular surveillance. Field detection of parasites in potential reservoir rodents and alternative vectors must be complemented by experimental validation of host and vector competence. Only through this combination of evidence can transmission cycles be reliably reconstructed, true sources of infection identified, and the risk of emergence in new regions anticipated.

Molecular studies reveal further unexpected features. *Mundinia* genomes are smaller than those of the *Leishmania* and *Viannia* subgenera and display a reduced repertoire of surface molecules, including amastins and LPG-modifying enzymes, that are critical for macrophage interactions and sand fly development [3]. In addition, *L. martiniquensis* harbors the first leishbunyavirus identified outside the *Leishmania* RNA virus (LRV) lineage [24]. Since LRVs from the Totiviridae family documented in the *Leishmania* and *Viannia* subgenera augment pathogenicity of these parasites [25], intriguing questions have arisen regarding potential effects of leishbunyavirus on biology and pathogenicity of *Mundinia*.

Despite these unusual characteristics, *Mundinia* parasites generally appear to coexist peacefully with their hosts, causing disease only sporadically. Most infections are asymptomatic, reinforcing the idea that these organisms are highly adapted to cryptic and finely balanced ecological niches. As such, they provide a valuable window into host–parasite co-evolution and challenge the assumption that pathogenicity and widespread transmission are inevitable hallmarks of medically relevant parasites.

Ultimately, the study of *Mundinia* expands our understanding of what it means to be a *Leishmania* parasite. These enigmatic organisms defy traditional expectations, reminding us that even well-characterized pathogen groups can harbor lineages that operate under different ecological and evolutionary rules. Recognizing and studying these exceptions is not merely an academic exercise: it has direct implications for One Health, the recognition of emerging diseases, and our capacity to predict pathogen behavior in a rapidly changing world. *Mundinia* remind us that the rules of leishmaniases are less fixed than once believed—and that some of the most informative parasites are those that refuse to conform.
